# Modeling of spatial distribution for scorpions of medical importance in the São Paulo State, Brazil

**DOI:** 10.14202/vetworld.2015.823-830

**Published:** 2015-07-07

**Authors:** José Brites-Neto, Keila Maria Roncato Duarte

**Affiliations:** 1 Epidemiological Surveillance Department, Secretariat of Health, Americana, São Paulo, Brazil; 2 Department of Genetics and Animal Reproduction, Institute of Animal Science, Nova Odessa, São Paulo, Brazil

**Keywords:** binomial probability, environmental variable, georeferencing, maxent, *Tityus bahiensis*, *Tityus serrulatus*

## Abstract

**Aim:**

In this work, we aimed to develop maps of modeling geographic distribution correlating to environmental suitability for the two species of scorpions of medical importance at São Paulo State and to develop spatial configuration parameters for epidemiological surveillance of these species of venomous animals.

**Materials and Methods:**

In this study, 54 georeferenced points for *Tityus serrulatus* and 86 points for *Tityus bahiensis* and eight environmental indicators, were used to generate species distribution models in Maxent (maximum entropy modeling of species geographic distributions) version 3.3.3k using 70% of data for training (n=38 to *T. serrulatus* and n=60 to *T. bahiensis*) and 30% to test the models (n=16 for *T. serrulatus* and n=26 for *T. bahiensis*). The logistic threshold used to cut models in converting the continuous probability model into a binary model was the “maximum test sensitivity plus specificity,” provided by Maxent, with results of 0.4143 to *T. serrulatus* and of 0.3401 to *T. bahiensis*. The models were evaluated by the area under the curve (AUC), using the omission error and the binomial probability. With the data generated by Maxent, distribution maps were produced using the “ESRI^®^ ArcGIS 10.2.2 for Desktop” software.

**Results:**

The models had high predictive success (AUC=0.7698±0.0533, omission error=0.2467 and p<0.001 for *T. serrulatus* and AUC=0.8205±0.0390, omission error=0.1917 and p<0.001 for *T. bahiensis*) and the resultant maps showed a high environmental suitability in the north, central, and southeast of the state, confirming the increasing spread of these species. The environmental variables that mostly contributed to the scorpions species distribution model were rain precipitation (28.9%) and tree cover (28.2%) for the *T. serrulatus* and temperature (45.8%) and thermal amplitude (12.6%) for the *T. bahiensis*.

**Conclusion:**

The distribution model of these species of medical importance scorpions in São Paulo State revealed a higher environmental suitability of these species in the regions north, central, and southeast of the state, warning to emergencies actions for prevention and surveillance from scorpion stings in several counties. There is also a need to best conservation strategies related to neighboring territories, with the implementation of new environmental protected areas and measures of spread control of these species in urban areas of several counties.

## Introduction

Scorpions (Order Scorpiones) are members of the Class Arachnida (Phylum Arthropoda, Subphylum Chelicerata) with 2000 species distributed worldwide except Antarctica, and most of these species occur in tropical and subtropical regions [1]. Among the 18 scorpion families described in the world, four families are reported in Brazil (Bothriuridae, Buthidae, Chactidae, and Hemiscorpiidae), with 23 genera and 160 species representing 9% of the world’s diversity [2]. We highlight the family Buthidae with 63% of scorpion’s species distributed in eight genera, and the genus *Tityus* C.L. Koch, 1836, with 54 species in all geographic regions and in different types of ecosystems [3].

Some species can easily adapt to anthropic environments and it is possible that an urban environment favors the development of new scorpion population [4] as is the case with Brazilian *Tityus serrulatus* Lutz e Mello, 1922, opportunistic species and parthenogenetic, with a reproductive strategy of colonization and proliferation in the cities and a determinant factor for propagation of scorpionism in Brazil, with registered population outbreaks, mainly in the northeast and southeast regions [5].

The urbanization contributes to global environmental change in various ways and at various dimensions [6]. Urban growth at our cities and anthropogenic derived changes in conservation areas and nature reserves facilitate the development of many wild species adapted to synanthropic environment [7], where the scorpions have become urban pests causing serious human accidents of medical importance [8] with an estimated annual global incidence of about 1.5 million envenoming involving 2600 deaths [9].

The vast majority of species have specific requirements with regard to habitat and micro-habitat and ecological and biogeographical patterns predictable and localized, but some species of the genus *Tityus* (the yellow scorpions, *T*. *serrulatus* and brown scorpions, *Tityus bahiensis* Perty, 1833) have high ecological plasticity and irregular distribution patterns and may even occur in disturbed or modified environments by man, where they find shelter and food inside and/or near human dwellings [10].

The scorpion envenomation is a major public health problem in Brazil, mainly due to rapid dispersal of species in urban areas and the high toxicity of its venom, where approximately 50,000 annual cases are reported in this country [11,12] with a high incidence in warmer climates [13]. Yellow scorpion *T*. *serrulatus* is considered the most dangerous species in South America, as an endemic species in Brazil and distributed in the States of Bahia, Espírito Santo, Minas Gerais, Rio de Janeiro, São Paulo, Paraná, Goiás, and the Federal District [14]. Due to the existence of appropriate environment for its proliferation, such species found ideal conditions for their maintenance and reproduction in São Paulo State [15].

A study of geographic distribution of species of medical importance (*T*. *bahiensis* and *T*. *serrulatus*) based on the notification of scorpion stings and occurrence of the species found, alone or together, in 323 municipalities of five regions in São Paulo State, encountered *T*. *bahiensis* in 79% (254/323) and *T*. *serrulatus* in 65% (210/323) of these [16].

The north, with 52% of species of scorpions in the country, highlighting the Amazonas State, with 38 species, and the northeast with 26% of the Brazilian scorpions, especially Bahia State, with 27 species, are mainly responsible for the high species richness of scorpions in Brazil [2], and probably, the regions of origin of dispersion of the main species of scorpions of medical importance, including the two species treated here.

Scorpions are well-adapted to urban environment producing different patterns of population dynamics when in comparison to natural habitats [17]. Plenty of scorpion species depend on factors such as rainfall, temperature, and prey availability, with higher population dynamics of the species *T*. *serrulatus* and *T*. *bahiensis* in warmer and rain period (September to March, in South Hemisphere). The abundant rainfall determines flooding areas where scorpions live, facilitating their dispersal to new shelters. *T*. *serrulatus* is highly adaptable to different thermal zones having a preference for temperatures between 14°C and 38°C, but also being able to tolerate lower temperatures [18]. Scorpions react positively to humidity and negatively to temperatures higher than 39°C and the light [19]. The temperature is the most limiting factor for the geographical distribution of scorpion’s species affecting their ability of osmoregulation, as well as their cuticle permeability [20]. There is a strong association between temperature and the scorpion’s activity [21].

The species distribution modeling are a promising field of research for setting priorities for further conservation studies, by building environmental suitability maps, based on geographic occurrence of the species and its correlation to environmental indicators, in order to obtain models that can provide high predictive success and from highly significance [22]. Maps have long been a useful tool for visualizing patterns in health surveillance and one tool that can be used to apply advanced geospatial methods to health care problems with the help of geographic information system (GIS) [23].

In this work, we aimed to develop maps of modeling geographic distribution correlating to environmental suitability for the two species of scorpions of medical importance (*T*. *serrulatus* and *T*. *bahiensis*) at São Paulo State, aiming to analyze the dispersion processes of these species and to sustained spatial configuration parameters for epidemiological surveillance of these venomous animals.

## Materials and Methods

### Ethical approval

This study was subject to the regulations of Public Policy and Ethics Health Commitee in Brazil, defined by the Unified Health System (SUS) and applied to the System Information and Notifiable Diseases (SINAN). Other information inherent in the study was obtained at the Access Databases and Public Domain.

### Study area and data source

In Brazil, São Paulo State has a land area of approximately 248,222 km² and a population exceeding 44 million inhabitants being the state with the highest urbanization in the southeast, with 4971 km² which part of urban perimeters in the 645 municipalities and a demography density of 177.4 inhab/km². It has varieties of climate subtropical, tropical, and tropical according to the altitude (Cfa, Cfb, Cwa, Cwb, Aw).

**Figure-1:**
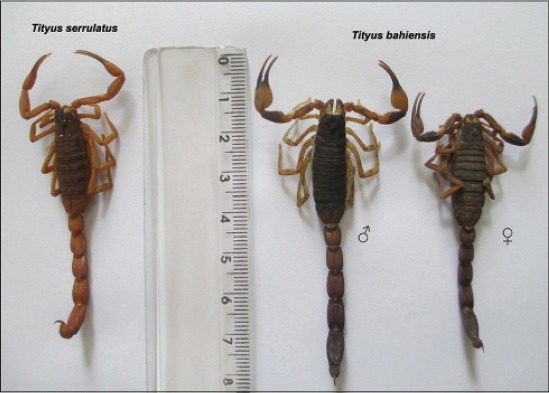
The parthenogenetic and sexed species, *Tityus serrulatus* and *Tityus bahiensis*, respectively.

**Figure-2:**
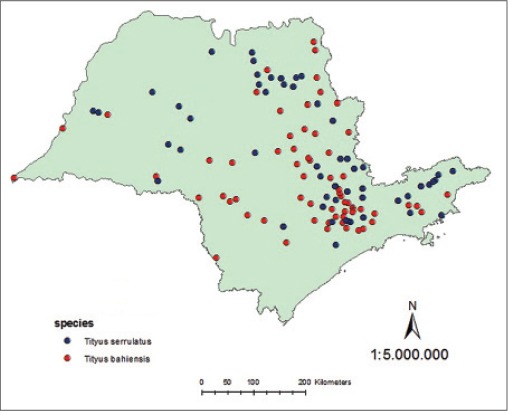
The scorpions species occurrence with georeferenced points at São Paulo State.

In this study, data of *T*. *serrulatus* and *T*. *bahiensis* (Figure-1) were obtained from 54 georeferenced points of *T*. *serrulatus* and 86 georeferenced points of *T*. *bahiensis* (geographic projection, WGS_1984_UTM_Zone_23S) at São Paulo State (central meridian 49°W Greenwich, parallel 20°10’S and 24°50’S), recorded in UNICAMP and SinBiota databases and available for download at “species link” (http://splink.cria.org.br/), based both in the occurrence of specimens as accidents, and mapped through the “ESRI^®^ ArcGIS for desktop 10.2.2” software (Figure-2).

Eight environmental indicators (altitude, annual average temperature, seasonality of temperature, annual temperature range, annual rainfall, seasonality of precipitation, drainage density, and percentage of tree cover) available for download at “AMBDATA” (http://www.dpi.inpe.br/Ambdata/) were used.

### Maxent software package

Even though the two species of scorpions treated here present different microenvironmental characteristics, the geographical point of view, and ecological, the environmental covariants in conjunction with scorpions occurrence data, were modeled using a maximum-entropy algorithm incorporated in the Maxent software package (maximum entropy modeling of species geographic distributions) Versão 3.3.3k [24]. Due to the robust performance related to other models [25] and its statistical validation [26] was possible to build species distribution modeling through a balanced risk-assessment and with an adequate reliability.

Maxent was used to model species distribution, since this algorithm results in more accurate and conservative models of distribution, giving fewer errors by extrapolation, and there is no need of absence data [26], which are difficult to obtain and have low reliability for the tropical region. This algorithm estimates a distribution more uniform (maximum entropy) within the area of analysis, taking into consideration the environmental information obtained from the records of the presence of the species [24] and has been indicated as the most efficient in predictive modeling of species distribution [25,27].

Maxent quickly processes data to create probability distributions based on environmental parameters and species occurrence to species distribution mapping, estimating the distribution across geographical space or the density in covariate space [28]. Predictive modeling has being set to models and techniques in a smaller scale that can provide a more explicit risk exposure for individuals [29].

### Modeling of species geographic distributions

The environmental indicators were used to generate species distribution models in Maxent (maximum entropy modeling of species geographic distributions) version 3.3.3k using 70% of data for training (n=38 to *T*. *serrulatus* and n=60 to *T*. *bahiensis*) and 30% to test the models (n=16 for *T*. *serrulatus* and n=26 for *T*. *bahiensis*) [30,31].

Data were sampled by bootstrap (method of random resampling with replacement) in 10 random partitions with substitutions. The format “logistics threshold output” was used resulting in continuous values for each grid cell on the map from 0 (inadequate) to 1 (most appropriate). Hence, it was possible to interpret as the probability of the presence of suitable environmental conditions for the target species [32].

The models were evaluated by the area under the curve (AUC), the omission error, and the binomial probability. The calculation of the AUC was done, which is a measure that summarizes the quality of the model to measure their predictive power for a variety of thresholds for conversion into the binary model [31]. The AUC is a measure of accuracy ranging between 0.5 to fully random models, and 1.0, for the models in which the data used to test the model were perfectly predicted [24]. With the data generated by Maxent, distribution maps were produced using the “ESRI^®^ ArcGIS 10.2.2 for desktop” software.

## Results

Within this scenario, eco-epidemiological with a very evident anthropogenic pressure, the models generated in this analysis had high predictive success (AUC=0.7698±0.0533, omission error=0.2467, and p<0.001 for *T*. *serrulatus* and AUC=0.8205±0.0390, omission error=0.1917, and p<0.001 for *T*. *bahiensis*) where AUC scores obtained were considered satisfactory (Table-1).

**Table-1:** Values of AUC, omission test, binomial probability, and thresholds resulting from Maxent.

Species	*Tityus serrulatus*	*Tityus bahiensis*
Test AUC	0.7698	0.8205
AUC standard deviation	0.0533	0.0390
Minimum training presence logistic threshold	0.1644	0.0731
10 percentile training presence logistic threshold	0.2306	0.2582
Maximum test sensitivity plus specificity logistic threshold	0.4143	0.3401
Maximum test sensitivity plus specificity test omission	0.2467	0.1917
Maximum test sensitivity plus specificity binomial probability	0.0003	0.0000

AUC=Area under the curve

In this analysis, the environmental variables that mostly contributed to the scorpions species distribution model were rain precipitation (28.9%) and tree cover (28.2%) for the *T*. *serrulatus* and temperature (45.8%) and thermal amplitude (12.6%) for the *T*. *bahiensis* (Table-2).

**Table-2:** Estimative of the relative contribution of environmental variables to the Maxent model.

Variable (*T. serrulatus*)	Percent contribution	Variable (*T. bahiensis*)	Percent contribution
Precipitation	28.9	Temperature	45.8
Tree cover	28.2	Thermal amplitude	12.6
Drainage density	11.2	Tree cover	10.2
Seasonality of precipitation	10.5	Drainage density	9.6
Temperature	10.4	Precipitation	9
Thermal amplitude	4.7	Seasonality of precipitation	7.2
Altitude	3.8	Altitude	3.1
Seasonality of temperature	2.3	Seasonality of temperature	2.6

T. serrulatus=Tityus serrulatus, T. bahiensis=Tityus bahiensis

**Figure-3:**
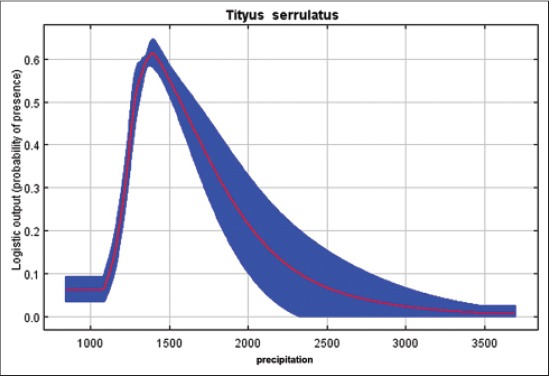
Probability of presence of *Tityus serrulatus* in the environmental variables of precipitation.

**Figure-4:**
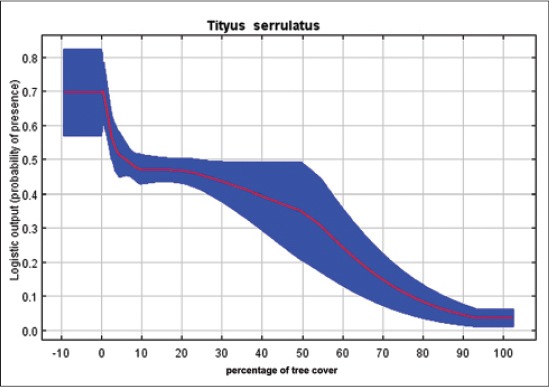
Probability of presence of *Tityus serrulatus* in the environmental variables of the percentage of tree cover.

**Figure-5:**
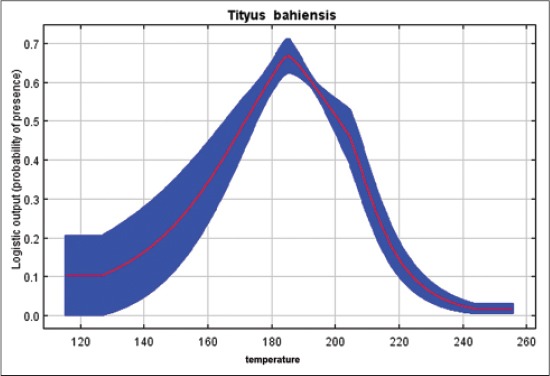
Probability of presence of *Tityus bahiensis* in relation to the environmental variables of temperature.

**Figure-6:**
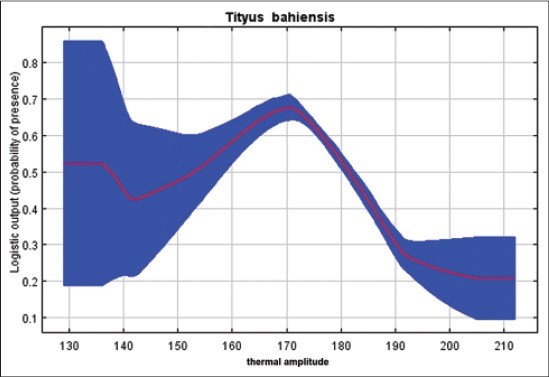
Probability of presence of *Tityus bahiensis* in relation to the environmental variables of thermal amplitude.

Our results indicated that *T*. *serrulatus* is very sensitive to the environmental variable of pluviometric precipitation, demonstrating that higher rates decrease the environmental suitability for this species, keeping up with good distribution between 1100 and 1800 annual mm^3^ (Figure-3). Regarding the percentage of tree cover, we find that for the case of a synanthropic species highly adapted to geographic regions with high anthropogenic interference, lower rates increase the probability of presence of this species of scorpions (Figure-4). *T*. *bahiensis* is very sensitive to environmental variables of temperature (Figure-5) and annual temperature range (Figure-6), demonstrating a major environmental suitability for this species, on the average ranges of values of these variables.

**Figure-7:**
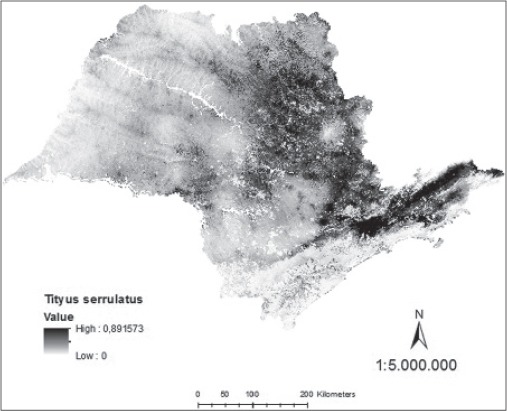
Model of continuous probability for *Tityus serrulatus* in the state of São Paulo.

**Figure-8:**
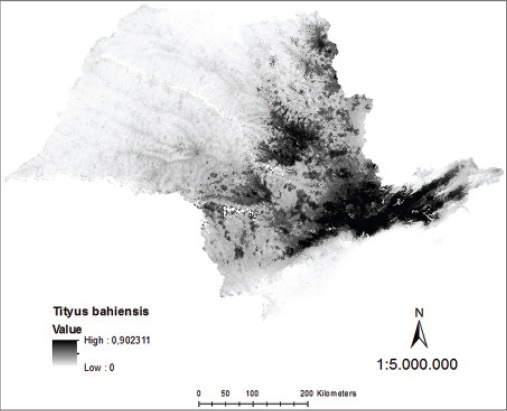
Model of continuous probability for *Tityus bahiensis* in the state of São Paulo.

Analyzing the map generated of probability continuous, it was verified the occurrence of distribution of *T*. *serrulatus* in northern and northeastern regions of São Paulo State, with the highest density in the metropolitan region of Campinas, in Greater São Paulo and Paraíba Valley (Figure-7). Regarding *T*. *bahiensis*, the map of continuous probability distribution indicates its occurrence in the northern, south-central, and northeastern of São Paulo State with the highest density in the metropolitan area of São Paulo and Paraíba Valley (Figure-8).

In this analysis, the “logistic threshold” used to cut models in converting the continuous probability model into a binary model was the “maximum test sensitivity plus specificity,” provided by Maxent, with results of 0.4143 for *T*. *serrulatus* and 0.3401 for *T*. *bahiensis* (Table-1).

**Figure-9:**
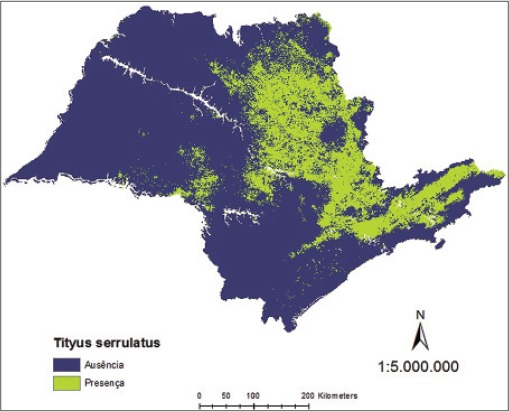
Binary model to *Tityus serrulatus* in São Paulo State.

**Figure-10:**
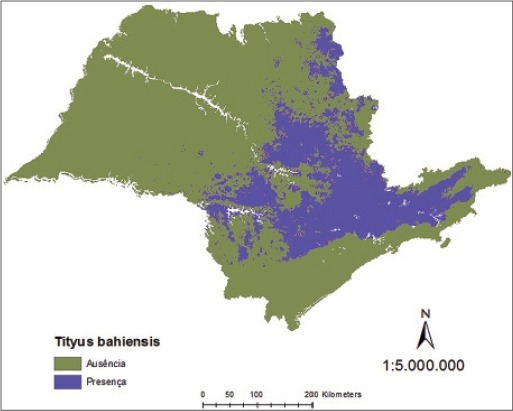
Binary model to *Tityus bahiensis* in São Paulo State.

**Figure-11:**
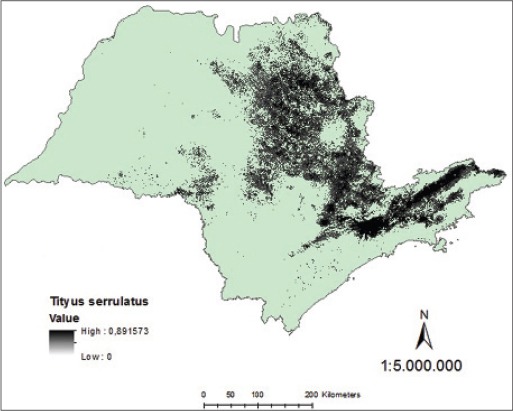
Final probabilistic model for *Tityus serrulatus* in São Paulo State.

**Figure-12:**
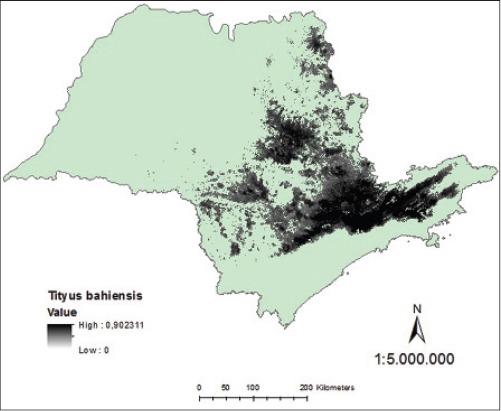
Final probabilistic model for *Tityus bahiensis* in São Paulo State.

Consequently, the binary map resulting for *T*. *serrulatus* and *T*. *bahiensis*, associated to environmental variables, shows a distribution of their presence more evident in the north and northeast regions of the São Paulo State (Figure-9) and in the north, south-central, and northeast of the São Paulo State (Figure-10), respectively, and the final probabilistic model for the species *T*. *serrulatus* and *T*. *bahiensis*, defines the areas of greatest risk of scorpion sting in accordance with their geographical distribution in major intensity in the northern and northeastern regions of São Paulo State (Figure-11) and in the northern, south-central, and northeastern regions of São Paulo State (Figure-12), respectively.

## Discussion

The lack of georeferenced data on scorpions in the context of public health, due to its condition characterized as a grievance neglected by most care and health surveillance services, makes it difficult the execution of tools applied in spatial modeling that can contribute to the interpretation of epidemiological reality on the populations at greater risk. At same time, the lack of ecological studies with these organisms as models in neotropical regions is not surprising, since no experts are available in Brazil, despite the high biodiversity of such environments and evidence that environmental change is transforming the ecology of the tropics [33]. It is known that the scorpion population in Brazil are underestimated due to lack of specialists and incentives for research, in addition to gaps in sampling of scorpions in several areas [2].

Any event in health can be influenced by the external environment and the relation between health and climate has become the focus of attention of health surveillance services, providing new opportunities for early detection, prediction, and prevention of adverse effects to public health [29].

GIS have proven to be a suitable approach for the analysis of environmental components which affect the spatial distribution of diseases [34]. The public health community has begun to use GIS data in combination with field surveys and census data to identify population at risk [6]. GIS is an automated system for the input, storage, analysis, and output of spatial information, whose applications comprise, forecast the epidemics, geographical distribution studies, and variation of diseases [35].

The techniques of remote sensing and GIS provide an analysis of the profile of environmental variables associated with incidence data of a health grievance or a disease [36] and their use to evaluate species distribution of vectors and plagues has evolved considerably with a modeling approaches using either logistic regression or discriminant analysis techniques to investigate associations between multivariate environmental data and patterns of presence or absence, on mapping of health risk [37]. The modeling methods are capable of predicting the probability of the presence of the dependent variable from a set of independent variables, being used to make risk maps from sample data sets based on the observed similarity of environmental conditions of sites [37]. The environment plays an important role in generating protected areas within endemic regions, but it could also contribute to the creation of hotspots with clusters indicating a high level of transmission or activity in comparison to neighboring areas [38].

The assessments of the biogeographic processes help understanding and explaining the current patterns of endemism and diversity in the neotropics where 24% of the global hotspots of biodiversity and Atlantic Forest are one of the top five priority areas for conservation (hottest hotspots), due to its mega-diversity and high vulnerability [39].

The parameters presented in this study showed how each environmental variable affects the Maxent prediction, indicating the variation on the logistic prediction changes in each environmental condition, maintaining all other environmental variables in their average sample value. The resulting graphics demonstrated the dependence of aptitude provided in both variable chosen as in the dependence induced by correlations between the selected variable and others variables, reflecting the average response of ten Maxent races replicated and the average plus or minus standard deviation. Since the predictive models generated have a continuous distribution of probability of the presence of the species, a conversion to a binary model of presence and absence is required for calculating the performance of the model [31]. The logistic threshold is an adaptive parameter of the logistic regression model that is used in classification models and evaluated using a logistic regression algorithm. From the successes and errors in the prediction of new presences and pseudo-absences were calculated by the algorithm itself, indices of predictive performance of the model: sensitivity (probability of a sample be correctly classified as presence) and specificity (probability of a sample be properly classified as absent).

Several biogeographical mappings [16,40-42] discussed and presented validations of hypotheses related to the processes of *T*. *serrulatus* dispersion from the center-south region of Bahia State in the northeast and along the east-central region of Minas Gerais State, in close association with areas of Cerrado, Caatinga, and some refuges of remaining areas of Atlantic forest. From an eco-epidemiological perspective, for these species, a influence of anthropogenic environments between road axis of the metropolitan areas of Salvador and Belo Horizonte can be admitted, mainly represented by highway Governor Mario Covas (BR-101) and adjacencies, besides some influence of environmental variables related to the watershed of the São Francisco River on the originating dispersion of these territories to São Paulo State.

Ours results obtained by Maxent to model species distributions, corroborate and contribute to the understanding that from Minas Gerais State, *T*. *serrulatus* was distributed along the Valley of Paraiba in Rio de Janeiro State and São Paulo State, with probable contribution from road and rail corridor along highway President Dutra (BR-116) and by environmental variables along the Rio Paraiba do Sul. From there, it was dispersed through the territory of the São Paulo State for the metropolitan regions of São Paulo and Campinas, probably through an intense flow of cargo by highway (SP-348 Bandeirantes highway, SP-330 Anhanguera highway) and railway (Ferroban), by the active interference of anthropogenic processes determined by the growing conurbation and intense urbanization between various cities and by deforestation of riparian forests of the watershed of the rivers Tietê, Atibaia, Capivari, Jaguarí, and Piracicaba with gradual destruction of forest fragments.

The individual differences in the use of the environment can facilitate coexistence among scorpion’s species. Competition for shelters at different spatial scales and predation pressure can highly affect the dynamics and distribution of scorpion species in a tropical region where intra- and inter-specific coexistence presents in several species of scorpions, different levels of aggregation, and sociability [33]. Parthenogenesis is of fundamental importance in the biology of scorpions, particularly for *T*. *serrulatus*. This reproduction mechanism plays an important role in spreading and establishment of new population of species of scorpions because through a single individual can generate a new population [3].

Regarding *T*. *bahiensis*, notoriously, the dispersion became more conditioned to their biological and ecological requirements, represented by a sexed reproduction and strong relationship with environmental variables of temperature and humidity, restricting their home range on the central and southwestern regions of Minas Gerais State, west of São Paulo State, and northern Paraná State. Its presence on the watershed of the rivers Paraná and Tietê, associated with soil moisture generated by groundwater that are part of the Guarani Aquifer complex in these regions with low human density and greater exuberance of forest fragments apparently have been characteristics of this species which choose refuges of ecological niche, in contrast to *T*. *serrulatus* that presents a synanthropic behavior more evidenced and related to anthropic processes.

It is possible that the modern means of transport (rail, road, and air) and human settlements in new areas, in association with the biological phenomena of parthenogenesis in *T*. *serrulatus*, are preponderant factors for the propagation of these arthropods [3,4] and that the presence of conspecific and hetero-specifics in the same environment most certainly results in substantial competition for the resources of food and shelter and may decisively influence on habitat selection and dispersion into home range [33].

## Conclusion

The distribution model of two species of medical importance scorpions in São Paulo State (*T*. *serrulatus* and *T*. *bahiensis*) revealed a higher environmental suitability of these species in the regions north, central, and southeast of the state, warning to emergencies actions for prevention and surveillance of scorpion stings in several counties. There is also a need to best conservation strategies related to neighboring territories, with the implementation of new environmental protected areas and measures of spread control of these species in urban areas.

## Authors’ Contributions

Data were collected and interpreted by José Brites-Neto, using software ArcGIS and Maxent. The manuscript was prepared jointly by José Brites-Neto and Keila Maria Roncato Duarte. Keila Maria Roncato Duarte participated in the review process incorporating valuable suggestions for improvement of the manuscript. All authors read and approved the final manuscript.
